# Digitalising Regional Induction for Junior Doctors in Mental Health and Learning Disabilities Department of Betsi Cadwaladr University Health Board

**DOI:** 10.1192/bjo.2024.454

**Published:** 2024-08-01

**Authors:** Jiann Lin Loo, Vinila Zachariah

**Affiliations:** Betsi Cadwaladr University Health Board, Wrexham, United Kingdom

## Abstract

**Aims:**

Induction training is a crucial part of starting work in a new organization as it orientates new staff to their work role and environment, which ensures that they can work safely and competently. Given the wide geographical area of North Wales, there is logistic difficulty to continue with face-to-face induction sessions for new junior doctors. A digital format for regional induction for new doctors from all sites was introduced in 2021. This virtual induction has dealt with the accessibility problem effectively. Nevertheless, there seemed to be some ongoing issues regarding organising the session with speakers due to overlapping clinical duties. Therefore, a quality improvement project has been initiated to improve the delivery of the sessions with minimal disruption to clinical duties. This paper is aimed to share the preliminary experience of the process of digitalisation of the induction programme.

**Methods:**

The pilot regional induction with the above changes was carried out on August 4, 2023 via Microsoft Team Meetings and was accessible to new starters from all three sites in North Wales. The sessions consisted of talks from consultants, the lead clinical pharmacist, the ST in psychiatry and clinical services/Rota coordinator. The induction was divided into morning and afternoon sessions. The participants consisted CTs in psychiatry, GPSTs, and FY trainees. The session was recorded and a pre-recorded session on history taking was introduced. Any queries about pre-recorded session were answered by the chair of session.

**Results:**

It was found that an estimated time saved per induction was 285 minutes with an overall saving for 3 inductions per year of 14.25 hours. The estimated cost saved (based on the lowest pay scale in NHS, £) was £151.13 with an overall saving for 3 inductions per year of £453.39. There were two Assessments of Teaching (AoT) and two Direct Observations of Non-Clinical Skills (DONCS) signed.

**Conclusion:**

Digitalising the regional induction helps to save both time and cost for the health board. It also reduces the risk of speakers in availability. Furthermore, the recording can be sent out early to all the JDs before they join MHLD, which can facilitate a quicker orientation into the new role. It is also a good opportunity for core and specialty trainees to achieve competencies for leadership and teaching.

